# A systematic review and meta-analysis comparing tumor progression and complications between radiofrequency ablation and thyroidectomy for papillary thyroid carcinoma

**DOI:** 10.3389/fonc.2022.994728

**Published:** 2022-11-30

**Authors:** Yuan-dong Sun, Hao Zhang, Hai-tao Zhu, Chun-xue Wu, Miao-ling Chen, Jian-jun Han

**Affiliations:** ^1^ Department of Interventional Radiology, Shandong Cancer Hospital and Institute Affiliated Shandong First Medical University and Shandong Academy of Medical Sciences, Jinan, China; ^2^ Zoucheng People’s Hospital, Jining, China; ^3^ Graduate School of Shandong First Medical University, Jinan, China

**Keywords:** papillary thyroid cancer, papillary thyroid microcarcinoma, radiofrequency ablation, meta-analysis, systematical review

## Abstract

**Background:**

Papillary thyroid cancer (PTC) is the most frequent thyroid cancers worldwide. The efficacy and acceptability of radiofrequency ablation (RFA) in the treatment of PTC have been intensively studied. The aim of this study is to focus on extra detailed that may influent for PTC or papillary thyroid microcarcinoma (PTMC).

**Materials and methods:**

We identified a total of 1,987 records of a primary literature searched in PubMed, Embase, Cochrane Library, and Google Scholar by key words, from 2000 to 2022. The outcome of studies included complication, costs, and local tumor progression. After scrutiny screening and full-text assessment, six studies were included in the systematic review. Heterogeneity was estimated using I^2^, and the quality of evidence was assessed for each outcome using the GRADE guidelines.

**Results:**

Our review enrolled 1,708 patients reported in six articles in the final analysis. There were 397 men and 1,311 women in the analysis. Two of these studies involved PTC and four focused on PTMC. There were 859 patients in the RFA group and 849 patients in the thyroidectomy group. By contrast, the tumor progression of RFA group was as same as that surgical groups [odds ratio, 1.31; 95% CI, 0.52–3.29; heterogeneity (I^2^ statistic), 0%, *p* = 0.85]. The risk of complication rates was significantly lower in the RFA group than that in the surgical group [odds ratio, 0.18; 95% CI, 0.09–0.35; heterogeneity (I^2^ statistic), 40%, *p* = 0.14].

**Conclusions:**

RFA is a safe procedure with a certain outcome for PTC. RFA can achieve a good efficacy and has a lower risk of major complications.

## Introduction

Although the incidence of thyroid cancer is highly ranked of all cancers in the world, the risk of mortality is not higher compared with that in the others (1–3). Thyroid neoplasm includes benign neoplasm of thyroid gland and malignant thyroid neoplasm. Papillary thyroid cancer (PTC) is the most common malignant thyroid tumor; it originates from follicular cells of thyroid gland and accounts for more than 80% of thyroid tumor cases (4, 5). In addition, papillary thyroid microcarcinoma (PTMC) belongs to PTC (6, 7). PTMC is a major subgroup of PTC and accounts for about 25%–30% of all thyroid tumor cases (8, 9). According to the World Health Organization criteria, the diagnosis of PTMC is confirmed if the tumor is ≤10 mm in diameter, whereas non-PTMC cases with a tumor diameter >10 mm are diagnosed with PTMC (10). The detection rate of asymptomatic thyroid nodules has greatly increased by the wide use of thyroid ultrasound and other imaging methods (11–13).

Thyroidectomy is the main treatment of traditional thyroid tumor (14). Currently, most of the guidelines still recommend thyroidectomy as the first-line treatment for those with PTC (15, 16). After surgery, most early-stage patients have an excellent prognosis (17–19). However, thyroidectomy carries potential risks of temporary or permanent recurrent laryngeal nerve paralysis, hypothyroidism, and hypoparathyroidism, and unsightly scarring (20–23). In areas of high morbidity and medical costs, this will place a significant burden on the health system (24). Globally, radiofrequency ablation (RFA) has been shown to be a safe and efficient treatment for solid tumors (25, 26). RFA showed a series of advantages over surgical resection, for example, minimized trauma, less invasive, faster recovery time, less pain, reduced post-operative analgesic drug use, little or no scarring, and shorter length of hospital stay (27–30). By carefully choosing the starting pathway, adjusting hotspot positions, limiting ablation parameters (i.e., ablation power and ablation time), and injecting buffer (often normal saline) to make liquid isolation, RFA will able to prevent nerve and tissue damage (31).

Although some studies support the efficacy of RFA on PTC or PTMC, contrasting results were described in different clinical settings (32, 33). Although those evidence appears compelling, many questions remain. To date, no extensive and systematic review exists to assess relevant clinical outcomes. With this background, we assessed the evidence about the tumor progression and complications between RFA and thyroidectomy for patients with PTC or PTMC by undertaking a meta-analysis and systematic review. Our analysis combines all individual patient data from as much as retrospective studies. Larger sample size may have contributed to the increase in test whether the RFA had any difference over thyroid surgery. It is hoped that this research will contribute to a deeper understanding of oncotherapy.

## Materials and methods

This systematic review was conducted as per the Preferred Reporting Items for Systematic Reviews and Meta-Analyses (PRISMA) guidelines. The primary research objective was to determine whether there is a significant difference in tumor progression with RFA and thyroidectomy. The secondary aim was to determine which techniques have higher therapeutic safety. This study was not applicable for an ethical review, and therefore, the Ethics Committee dropped approval of this study after review.

### Literature search

A systematic literature search was carried out by two reviewers using PUBMED, Web of Science, Embase, Cochrane Library, and CNKI databases from January 2000 to July 2022, including the following terms: radiofrequency ablation, RFA, thyroidectomy, papillary thyroid carcinoma, papillary thyroid microcarcinoma, and thyroid, thyroid cancer. Then, the researchers used Google Scholar for a supplementary search. We did not impose any language restriction in the search nor contact any author for additional or unpublic information. To expand our search range as much as possible, reference lists of the retrieved articles were also screened for more data, and further search was performed on the basis of the existing results. Both prospective and retrospective studies were included. Conference abstracts were excluded.

Relevant studies were identified sequentially by the titles and abstracts screened and full‐text browsing by three reviewers. All uncertainties and controversial issue were resolved by consensus by re‐checking sources. In addition, the conformity of data to the inclusion and exclusion criteria for this study was resolved in the same way.

### Eligibility criteria

The criteria for inclusion of studies were as follows: (i) prospective or retrospective articles without ethical issues; (ii) the research content is consistent with our research topic; (iii) the comparative study of RFA and thyroidectomy; and (iv) the required data results should be reported from the article or can be derived.

Exclusion criteria were as follows: (1) article review, editorials, case reports, and letters; (2) the data contained in the article are duplicated; (3) studies using animal models (such as swine and rat) or unrelated to the subject of our theme; (4) mass missing data or insufficient data; and (5) when the same study was reported twice, we extracted data from the most recent study with the largest sample size for results.

On the basis of the evaluation criteria of the Cochrane Handbook, the included studies are evaluated and graded. The assessment items included (1) sequence generation, (2) allocation concealment, (3) no statistical difference between the two groups, (4) complete outcome data, (5) no selective outcome reporting, and (6) other sources of bias.

The methodological quality of the included studies was assessed using the six evaluation indicators on which three consequences of each eligible study were evaluated: “high risk”, “low risk”, and “not clear”. According to the authors, the majority of patients were randomly assigned to the enrollment group. However, the characteristic differences between surgery and RFA did not allow for a double-blind clinical trial.

### Data extraction

Study characteristics and results of the enrolled studies were extracted by two reviewers including the name of the first author, publication year, country or region, study design, sample size, gender, age, number of tumor recurrence cases, and complications. All data were extracted by one reviewer and checked by another one.

### Statistical methods

The statistical analysis was performed using Stata version 15 (Stata Corp, College Station, TX, USA) and Review Manager [RevMan (computer program), version 5.3; Copenhagen: The Nordic Cochrane Centre, The Cochrane Collaboration]. The funnel plot analysis and sensitivity analysis were carried out using R software version 4.2.0 (available at http://www.R-project.org/).

Data were pooled using odds ratios. A fixed-effects model was used. Statistical heterogeneity between studies was examined utilizing the χ^2^‐test and the I^2^ statistic. Cochrane stipulates that 0%–40% is mild heterogeneity, 40%–60% is moderate heterogeneity, 50%–90% is relatively heterogeneous, and 75%–100% is highly heterogeneous.

## Results

### Identified studies

**Figure 1:**
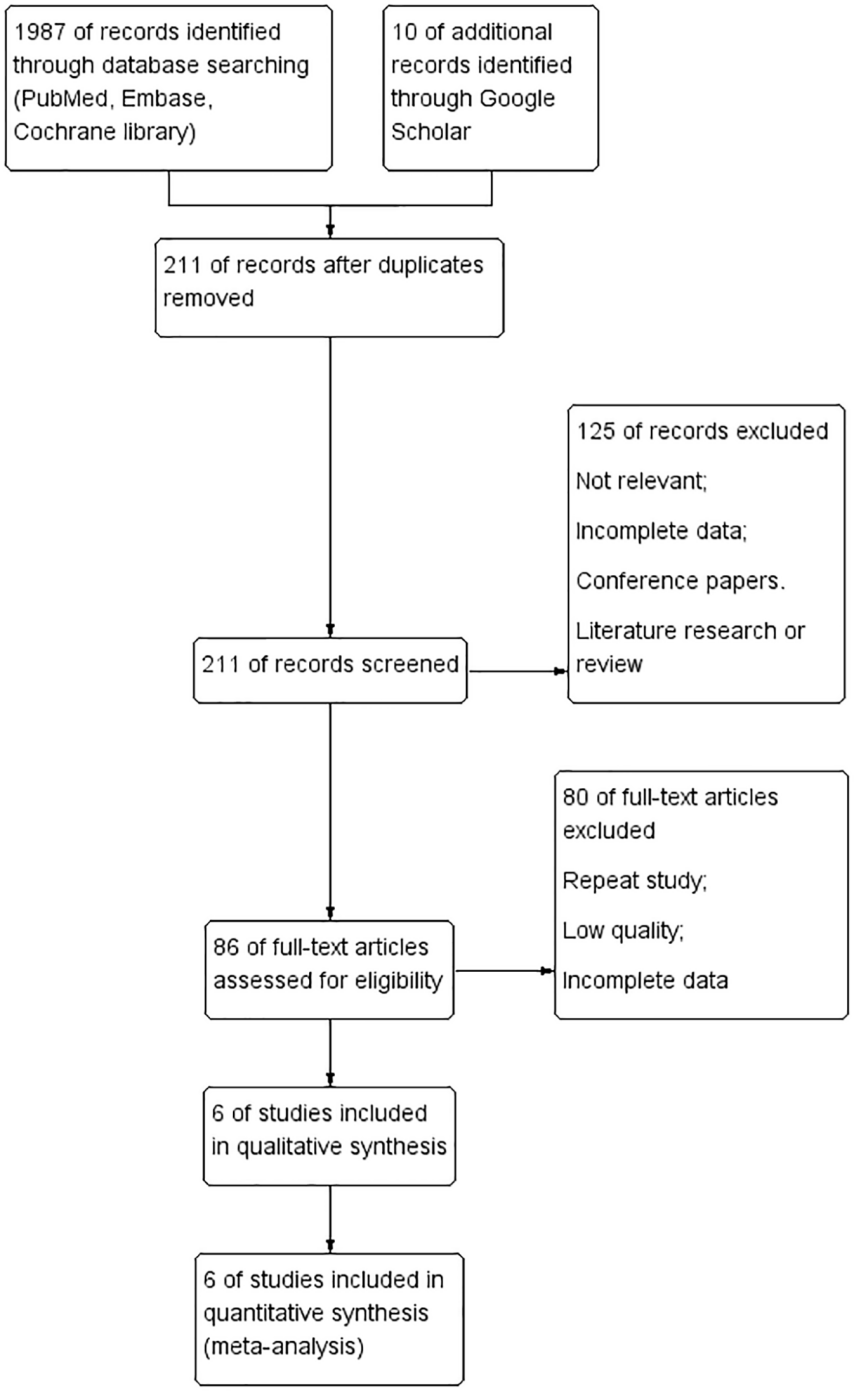
The Preferred Reporting Items for Systematic Reviews and Meta-Analyses (PRISMA) flow chart of the study selection process, showing the number of studies excluded at each step and the reasons for exclusion from the systematic review and meta-analysis.

A flow chart summarizing the study selection process is shown in **Figure 1**. From the initial search, 1,987 records were identified (**Figure 1**), of which 211 were selected for the title and abstract screening after removing duplicates. Of these, 125 were excluded, leaving 86 full-text articles for review. In addition, prospective cohort or retrospective studies that were a single group were excluded, leaving 80 articles. Six papers in total were ultimately retained for the present review.

A total of six studies were identified (34–39). Details of these studies are shown in **Tables 1**, **2**. All of studies were conducted in China. Four of the studies are about PTMC, and others are about PTC. All included studies were retrospective studies that were published in English after 2013. Moreover, two studies are focused on earlier stage of PTC (T1M0N0). Practitioner blinding (patients and researchers) cannot be achieved because of the unique nature of the treatment.

**Table 1:** Baseline characteristics of the systematic review.

Author, year	Design	Country	Tumor type	PSM	Tumor stage	Time of enrollment
He, 2021	Retrospective	China	PTC	No	T1bN0M0	2014–2019
Lin, 2021	Retrospective	China	PTMC	Yes	NR	2014–2018
Song, 2021	Retrospective	China	PTMC	No	NR	2014–2018
Xiao, 2020	Retrospective	China	PTC	No	T1bN0M0	2014–2019
Zhang, 2019	Retrospective	China	PTMC	No	NR	2013
Zhang, 2021	Retrospective	China	PTMC	Yes	NR	NR

PTC, papillary thyroid carcinoma; PTMC, papillary thyroid microcarcinomas; PSM, propensity score matching; NR, not reported.

**Table 2:** Patients’ characteristics of the systematic review.

Author, year	Gender(F/M)	RFA			Thyroidectomy			Follow-up (months)
		Mean age (years)	Sample size (n)	Mean volume (cm^3^)	Mean age (years)	Sample size (n)	Mean volume (cm^3^)	
He, 2021	164/40	43.940	94	0.795	43.790	110	0.833	12
Lin, 2021	498/166	44.100	332	0.092	43.800	332	0.086	>48
Song, 2021	181/37	44.900	115	0.182	45.400	103	0.198	>24
Xiao, 2020	140/42	40.700	91	0.730	40.200	91	0.840	>12
Zhang, 2019	130/44	45.400	94	0.176	44.100	80	0.133	>60
Zhang, 2021	198/68	45.770	133	0.058	45.680	133	0.062	>12

RFA, radiofrequency ablation.

### Meta-analysis

**Figure 2:**
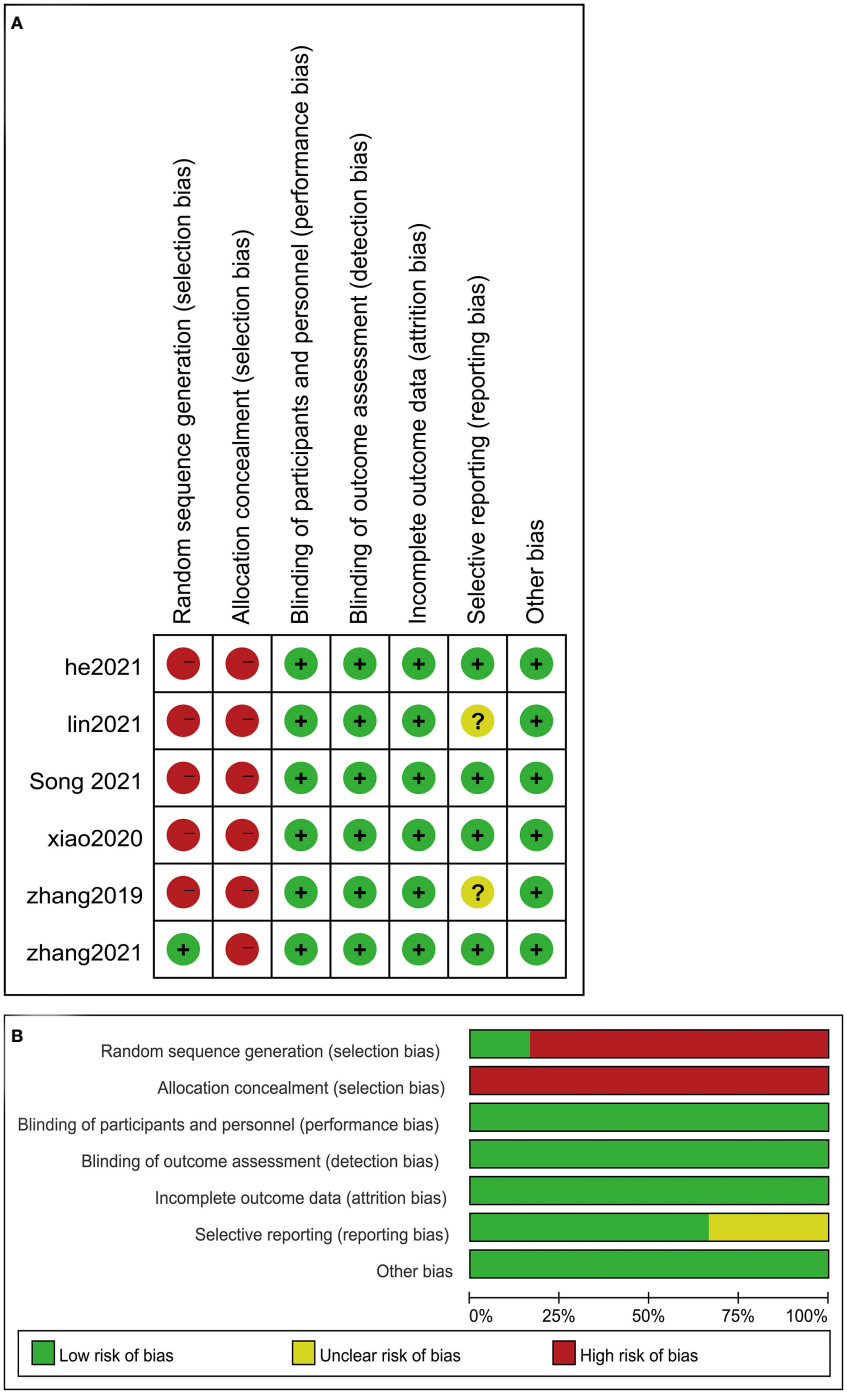
Risk of bias assessed according to the methods recommended by the Cochrane Collaboration. **(A)** Risk of bias graph: review authors’ assessment of risk of bias for each item presented as percentages across all included studies. **(B)** Risk of bias summary: review authors’ assessment of risk of bias for each item for the included studies. Question mark, unclear risk of bias; negative sign, high risk of bias; positive sign, low risk of bias.

Patients demonstrated significant reduction in the size of thyroid nodules after surgery or RFA treatment. It is evident that the risks of adverse effects associated with the treatments are small (**Figure 2** and **Table 3**). RFA shows a significantly smaller risk of complication (OR = 0.18, 95% CI = 0.09–0.35, P = 0.14). As for any treatments, the risk of tumor progression is minimal (OR = 1.31, 95% CI = 0.52–3.29, P = 0.85).

**Table 3:** Comparison of complications between the two groups.

Author, year	RFA complication						Thyroidectomy complications					
	Choking	DN	Fever	Numbness	Hoarseness	Hypothyroidism	Choking	DN	Fever	Numbness	Hoarseness	Hypothyroidism
He, 2021	0	0	1	0	1	0	3	1	1	2	6	0
Lin, 2021	0	0	0	0	0	0	0	0	1	0	12	2
Song, 2021	0	0	0	0	2	0	0	0	0	0	0	103
Xiao, 2020	0	0	0	0	0	0	0	0	0	0	0	4
Zhang, 2019	0	0	0	0	0	0	0	0	0	0	2	1
Zhang, 2021	0	0	0	0	2	1	0	0	0	0	4	1

RFA, radiofrequency ablation; DN, dizziness and nausea.

### Risk of bias

**Figure 3:**
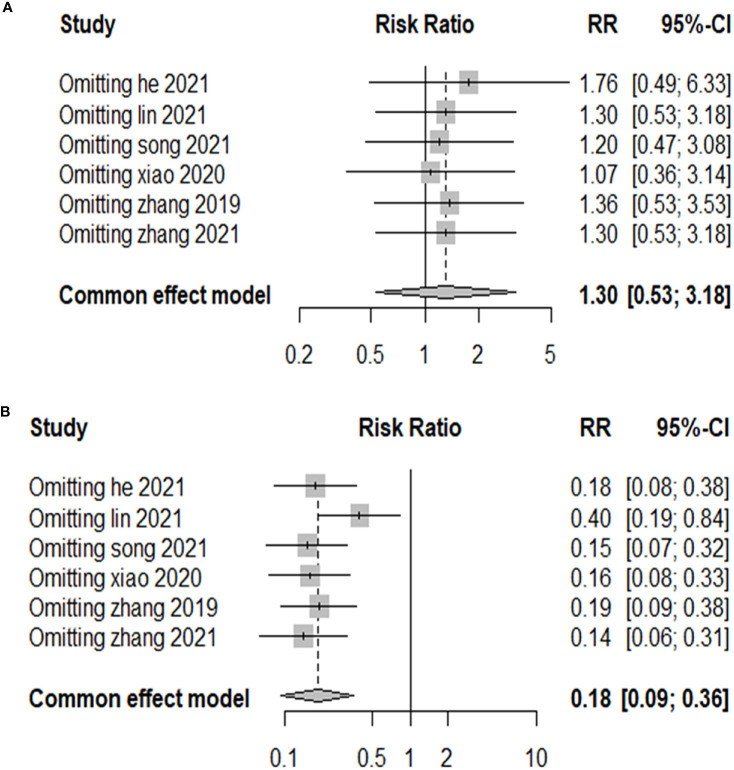
Risk of bias assessed according to the methods recommended by the Cochrane Collaboration. **(A)** Risk of bias graph: review authors’ assessment of risk of bias for each item presented as percentages across all included studies. **(B)** Risk of bias summary: review authors’ assessment of risk of bias for each item for the included studies. Question mark, unclear risk of bias; negative sign, high risk of bias; positive sign, low risk of bias.

The included studies had, in general, low or unclear risk of bias (**Figure 3**). Two most obvious characteristics were the lack of random sequence generation and allocation concealment and insufficient information in the articles to assess whether the patient assignment was random and hidden. In fact, true blinding (patients and researchers) cannot be achieved because of the unique nature of the treatment.

**Figure 4:**
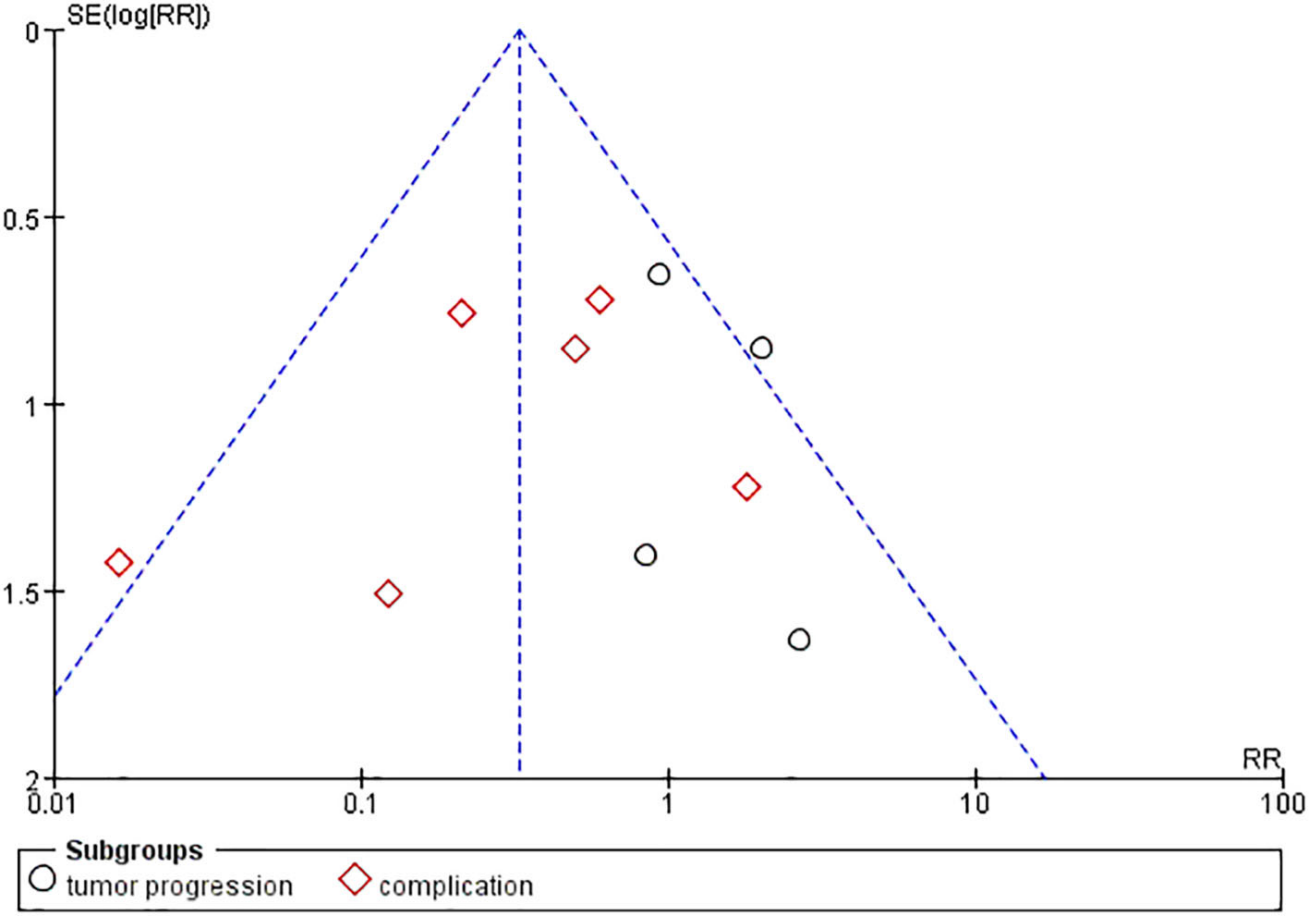
Funnel plots with pseudo 95% CIs for assessing the publication bias of the included studies. Standard Mean Difference (SMD) was plotted against SE for thyroid nodule size.

**Figure 5:**
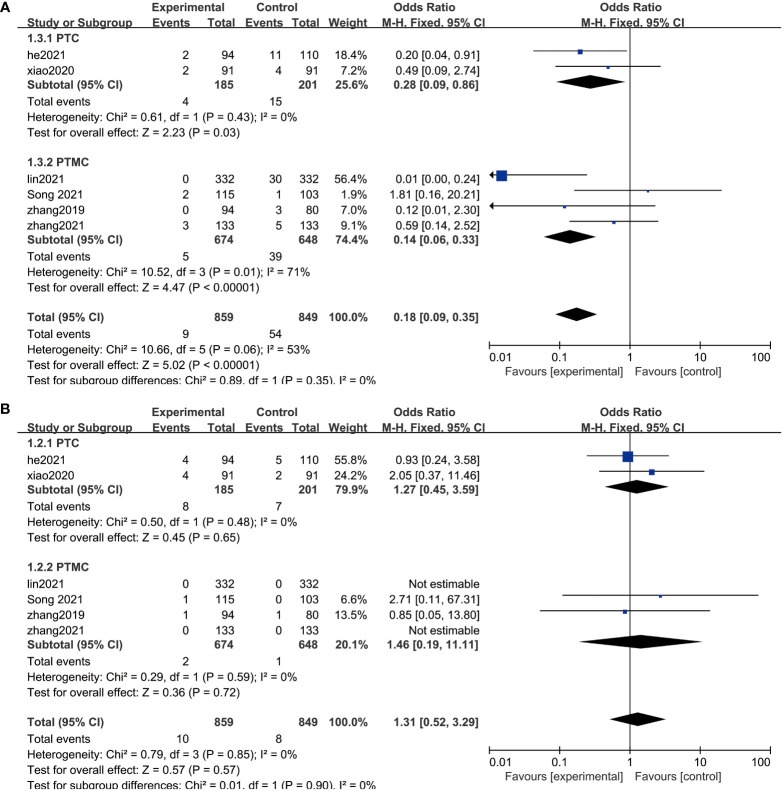
Sensitivity analysis. **(A)** Sensitivity analysis of the comparison of the efficacy in the two groups. **(B)** Sensitivity analysis of the post-treatment complications in the two groups.

Publication bias was examined using funnel plots (**Figure 4**) and was tested by performing Egger’s test. Our analysis revealed no evidence of publication bias. Two sensitivity analyses were performed to examine the robustness of our results. The results did not show obvious difference for the main outcomes (**Figure 5**).

### Subgroup analyses

PTMC is the term given to PTC that is 1 cm or less in diameter. To prevent the possible impact of the subtyping on the results, therefore, we performed a subgroup analysis by dividing them into two groups, PTC and PTMC, according to lesion diameter. After subgroup analysis, the results of meta-analysis did not show abnormal changes, which illustrated that RFA is safe and has a low risk of recurrence between PTC and PTMC (**Figure 5**).

## Discussion

RFA is a technically workable treatment method for malignant or non-malignant thyroid nodules, with no hypothyroidism effect or serious tissue damage of the normal thyroid tissue next to the heated zone (40). However, concerns about safety and long-term results of RFA, especially recurrence of tumor, persist. However, in our study, the results showed no significant difference between RFA and surgery in complication and tumor recurrence. In fact, it is the opposite. Some of the findings of our study are inconsistent with the previous research studies; the post-treatment recurrence was not expected to be higher in the RFA groups. There is no statistical difference in the short-term treatment results achieved between surgery and RFA, but there is a difference between the adverse effects between the two. RFA can show some advantages by virtue of its percutaneous puncture rather than surgical treatment.

Because of the specific biological behavior of thyroid tumors, there is an ongoing debate over whether the thyroid is overtreated rather than underappreciated. The American Thyroid Association (ATA) guidelines do not recommend biopsy for lesions under 1 cm with high ultrasound (US) suspicion of PTC and use a more conservative hemitotal resection rather than total resection for tumors under 4 cm in diameter (41). The ATA and NCCN thyroid cancer guidelines recommend hemithyroidectomy as an acceptable surgical treatment option for low-risk thyroid cancer. The treatment guidelines recommend subtotal thyroidectomy for patients with differentiated thyroid cancer greater than 1 cm in diameter or for patients with multiple lesions or metastases (42, 43). The Chinese expert guidelines on thyroid tumors state that overdiagnosis and overtreatment of PTMC due to widespread use of ultrasonography are of concern (44). Furthermore, the expert consensus on the diagnosis and treatment of micropapillary thyroid cancer in China published in 2016 acknowledges that there is controversy as to whether intra-glandular PTMC (especially those less than 5 mm in diameter) can be followed closely without surgery (45). It is undeniable that there is controversy among experts from various countries and organizations regarding the management of small-diameter thyroid tumors, but the recommendations for close follow-up are consistent. The controversy over therapeutic approaches and overtreatment also represents a need for therapeutic applications of RFA in this field.

RFA is a safe procedure that can be well tolerated by the patients and has low risk of complications. In all studies, there was no occurrence of a major complication or treatment-related death in the two groups. We could confirm that the ultrasonographic/CT-guided percutaneous RFA performed under local anesthesia is safe (46, 47). Pre-treatment clinical and medical imaging evaluations serve as the most important diagnostic modalities for determined suspected lesions. The TNM stage was confirmed and diagnosed before treatment by either physical examination, imaging, diagnostic tumor markers, or thyroid function test (48–50). Surgical treatment options exist with inevitably greater trauma and more tissue resection (51). Clinicians need to operate resections based on laboratory tests and imaging examinations data (52). RFA does not need skin incision or dissection and excision of thyroid tissue. The ability of RFA to provide real-time image guidance during treatment may also be a major reason for the lower number of complications (53, 54). It is generally considered that thyroidectomy is a safe surgery, but its complications are still common. Thyroidectomy is inevitably accompanied by bleeding and scarring, but there are advantages to RFA in terms of post-operative fever and nerve damage. Post-operative bleeding remains one of the most customary and fatal complications in thyroid surgery (55, 56). Furthermore, nerve palsy (recurrent laryngeal nerve), hypothyroidism, and hypoparathyroidism are the most common surgical complications of thyroidectomy (57–59). It should be noted that the risk of fatal complications after surgery is higher than that after RFA. Bleeding and post-operative hematoma can be a potentially fatal complication of thyroidectomy, because a rapid expansion of hematoma can cause tracheal compression, which can lead to breathing difficulty, asphyxiation, and death (60, 61). In contrast, skin burns and nerve injury are the most common complications in monopolar RFA treatment (62, 63). Comparatively, after RFA, the risk of asphyxia due to compression of the trachea is low.

A more important finding from the present study is that RFA can achieve long-term effects similar to thyroidectomy. RFA has been used widely in the treatment of solid cancers a long time ago. However, there are still challenges in determining the therapeutic effects of RFA in malignant thyroid nodules. All the patients in six studies were followed up for minimum of 1 year. At 1- to 5-year follow-ups of studies, the rate of tumor recurrence was not statistically different between the two groups. The between-group contrast analyses did not show any significant result. The rate of local tumor recurrence is low in the medium-term to long-term follow-up in RFA and thyroidectomy. For a conservative hypothesis, ablation coverage of RFA may be restricted to a very limited range of tissues, with a greater risk of recurrence (64). In terms of comparisons of surgical recurrence rates, local control, and disease-free survival, however, we were not able to make detailed comparisons with a high degree of confidence due to a lack of detailed data. However, in our analysis, results showed that there were no significant differences in the risk of tumor recurrence between this RFA and surgery. Furthermore, in those literature studies not included in our study, it was shown that thermal ablation, including RFA and microwave ablation (MWA), did not exhibit a higher risk of recurrence in the treatment of thyroid tumors, and no findings showed that RFA had a large difference from other surgical treatments in the long-term follow-up of treatment efficacy (65–67). In 2016–2019, we have performed approximately 150 cases of US-guided RFA or MWA treatment for PTC/PTMC in our center. In the earliest cohort of patients with 5 years of follow-up data (approximately 50 cases), none recurrence of the tumor was reported. At the same time, there were no reports of dissatisfaction or inconvenience with thermal ablation treatment. Thus, we point out that RFA does not associate with a greater risk of local tumor recurrence; at least there is no evidence of it at this time. Treatment protocol was not associated with recurrence of PTC. The results of this study are consistent with those of previous studies. This indicates that RFA may be a promising alternative to thyroidectomy for localized PTC (32). Therefore, some have questioned whether the removal of the thyroid gland, to be completely radical, leads to unnecessary damage. The extent of thyroidectomy for low-risk PTC is still debated (68–70). However, in most areas, total thyroidectomy or radical thyroidectomy still is the main surgical management for PTC or PTMC; it is the preferred approach for low-risk PTC (71, 72). Through the observations on the trait of the associated thyroid malignancy, PTC/PTMC is the most common endocrine malignancy usually with an indolent nature (73–75). As a result, some people question whether removing the whole or most thyroid gland for a radical cure target could cause some unnecessary damages (15, 76, 77). Thus, RFA was found to be a minimally invasive and efficient treatment method in the present study. For those patients who are intolerant of or unwilling to undergo thyroidectomy, undoubtedly, RFA for PTC/PTMC lesions under the help of medical imaging technical is a new treatment method. In addition, many patients have chosen RFA as an alternative option for aesthetics to avoid the complication or due to other reasons. In our meta-analysis, no differences between treatment effects were observed between both the PTMC and PTC subgroups, suggesting that RFA is reliable for the precise treatment of small and difficult-to-locate tumors.

Compared with surgery, RFA may offer some theoretical potential advantages. First, RFA is easy to learn, use, and master the technique; has a low risk of complications; and can be safely performed as an outpatient procedure (78, 79). Second, RFA do not need surgical removal, may save considerable cost and pre- and post-operative stay time, and reduce patient’s fear and anxiety perioperatively (80–82). Third, RFA has a protective effect on the patients’ facial appearance and maintenance of normal thyroid function, making it very attractive to young people and appearance-conscious patients. Last, the cost of RFA is significantly lower than that of surgical treatment, and given the high incidence of thyroid tumors in both developing and developed countries, there are undoubtedly significant savings in government health care expenditures (83).

RFA also has some disadvantages compared with other surgical procedures. First, RFA does not address the issue of occult PTMC lesions. Although the impact of occult lesions on the overall survival is minimal in low-risk thyroid tumors, they are still a potential risk (84). In addition, as the success of the ablation procedure is highly dependent on the experience of the operator and the post-ablation imaging changes, it should be performed by an experienced and senior physician to maximize the outcome of the treatment (85). Moreover, RFA treatment carries the risk of needle tract tumor seeding. Although the possibility of tumor metastasis after needle access adjustment is low, there are risks that cannot be ignored (86, 87). Fortunately, with the standardization of technology and more attention being paid to the risks of tumor metastasis, needle tract tumor seeding after RFA treatment is very rare. In our actual operation and some large-scale research studies, reports about it are not found. Finally, the negative impact on affecting histological diagnosis is another potential disadvantage of RFA, where needle biopsy after RFA cannot completely exclude the risk of invasive histological variants (88, 89). In recent clinical studies, researchers have quested that inadequate cytoarchitecture caused by coagulative necrosis may reduce the diagnostic accuracy of histological in the ablated zone (35). However, in the feedback that we received from the Pathology Department, ablation followed by biopsy will not have a significant negative impact on one’s pathological results (including qualitative diagnosis and immunohistochemistry).

This study has certain limitations. First, selection bias could not be completely excluded as all six studies in the analysis were retrospective. In addition, there was no random treatment allocation process in the allocation of the two groups of patients. Second, all articles are single-center studies, and we need multi-center, large-scale data to validate our analysis results. Third, the majority of patients included in the analysis of this study had insufficient follow-up time, considering the standard 5-year follow-up time for tumors.

In conclusion, the results of this study suggest that clinical outcomes of RFA for low-risk PTC/PTMC can be similar to those of surgical resection. As a safe and minimally invasive modality, RFA may be a promising alternative to existing PTC/PTMC management options.

## Data availability statement

The original contributions presented in the study are included in the article/supplementary material. Further inquiries can be directed to the corresponding author.

## Author contributions

Guarantors of integrity of entire study: Jj-H and Yd-S; study concepts/study design, data acquisition, and data analysis/ interpretation: H-Z, Ht-Z,Cx-W, and Ml-C; manuscript drafting and manuscript revision for important intellectual content: all authors; approval of final version of submitted manuscript: all authors; agreement to ensure any questions related to the work are appropriately resolved: all authors.

## Funding

This item was supported by the National Key Research and Development Program (No.2018YFE0126500), the Natural Science Foundation of Shandong Province (No. ZR2021MH060), Plan for Science and Technology Development of Ji’nan (No.201907124) and the Start-up fund of Shandong Cancer Hospital (2020-PYB20). The funding bodies played no role in the design of the study and collection, analysis, and interpretation of data and in writing the manuscript.

## Acknowledgments

The authors gratefully acknowledge all participants.

## Conflict of interest

The authors declare that the research was conducted in the absence of any commercial or financial relationships that could be construed as a potential conflict of interest.

## Publisher’s note

All claims expressed in this article are solely those of the authors and do not necessarily represent those of their affiliated organizations, or those of the publisher, the editors and the reviewers. Any product that may be evaluated in this article, or claim that may be made by its manufacturer, is not guaranteed or endorsed by the publisher.

## Abbreviations

PTC, Papillary thyroid carcinoma; PTMC, Papillary thyroid microcarcinoma; RFA, Radiofrequency ablation.
